# Agency and role models: do they matter for adolescent girls’ sexual and reproductive health?

**DOI:** 10.1186/s12905-023-02659-8

**Published:** 2023-09-27

**Authors:** Bolatito O. Ogunbiyi, Sarah Baird, Jeffrey B. Bingenheimer, Amita Vyas

**Affiliations:** 1 Department of Prevention and Community Health, Milken Institute School of Public Health, George Washington University, Washington, DC USA; 2 Department of Global Health, Milken Institute School of Public Health, George Washington University, Washington, DC USA

**Keywords:** Agency, Role models, Sexual and reproductive health, Adolescent girls, Ethiopia

## Abstract

Despite recent declines in early childbearing in Ethiopia, improved sexual and reproductive health continues to elude many adolescent girls, partially due to constrained agency and role models. This study examined the relationship between agency, role models and two sexual and reproductive health outcomes, ideal age at childbirth and attitude towards gender-differentiated parental control, among adolescent girls in Ethiopia. Agency and role model presence were positively associated with ideal age at childbirth (β = 0.23, p < .01 and β = 0.77, p < .001, respectively). Having family members, friends or famous individuals as role models was significantly associated with an increase of 1.45 years (p < .01), 1.32 years (p < .05) and 1.01 years (p < .01) in ideal age at childbirth, respectively, compared to having no role model. Agency was positively associated with attitude towards gender-differentiated parental control of adolescent behaviors (OR = 1.18, p < .001). This study highlights the need for interventions aimed at increasing agency and providing role models for adolescent girls.

**Supplementary Information:**

The online version contains supplementary material available at 10.1186/s12905-023-02659-8.

## Introduction

Adolescents aged 10–19 years currently make up about 16% of the world’s population – of the 7.9 billion people worldwide, 1.3 billion are adolescents [1]. Nearly 90% the global population of adolescents live in low- and middle-income countries (LMIC) [2]. In Ethiopia, adolescent girls aged 10–19 years make up over 12% or 12.6 million of the population, which is estimated to be over 120 million [3]. Despite constituting almost half of the adolescent population, little attention has been focused on specific challenges facing adolescent girls, including their sexual and reproductive health (SRH) needs, as they transition to adults, until recently [4].

For many girls in low- and middle-income countries (LMICs) such as Ethiopia, adolescence is a period of heightened vulnerabilities in terms of their sexual and reproductive health as it often coincides with dropping out of school, being married off very young, initiation of sexual activities, and unwanted pregnancy [4, 5]. More than half of Ethiopian girls aged 15–19 years are sexually active and about a third have started having children during this period [6–8]. The median age at marriage is 16.5 years and 40.3% of women aged 20–24 years are married before the age 18 [6]. These statistics reflect critical challenges to SRH of adolescent girls in Ethiopia and the need to address these issues. The socio-economic and cultural diversity of the country means that these national averages often mask the marked sub-national variations in critical SRH measures. For example, early childbearing ranges from 9.6% in Addis Ababa to 59% in Benishangul-Gumuz [7], indicating the need for targeted (e.g., region-specific) adolescent SRH interventions to address early childbearing. Furthermore, the distinct sociocultural diversity across different regions of the country implies that the experience of girls varies across these contexts.

In recent years, Ethiopia has experienced encouraging changes in social norms such as a decline in child marriage and early childbearing [9, 10]. Despite these improvements, optimal SRH outcomes continue to elude many adolescent girls in Ethiopia, and they remain the most vulnerable youth population in the country due to constrained choices, gender norms and resources [11]. The entrenched social norms such as early marriage and prioritization of boys in education diminish the ability of adolescent girls to make the right choices at the right time and are impediments to improving their sexual and reproductive health [12–14]. In addition, girls in Ethiopia have fewer opportunities than boys to interact with peers and role models, both of which can serve as positive forces in improving their SRH outcomes [15, 16].

### Gender attitudes: antecedents of SRH Behaviors among adolescent girls

While it is important to understand SRH behaviors among adolescent girls from a program planning perspective, extant literature suggests that measures of actual SRH behaviors (such as age at first sexual intercourse) that are usually explored in SRH studies among older adolescents and young women (15–24 years) may not be appropriate in studies that include very young adolescents (10–14 years) due to limited observations of sexual initiation among this group [5].

Since beliefs and attitudes towards future behaviors are formed during adolescence [17], it is more useful to understand the antecedents of SRH behaviors among adolescents such as gender attitudes before the outcomes manifest. These antecedents include ideals and attitudes towards a range of short and long-term sexual behaviors which may better reflect the social norms that later shape the SRH behaviors they later adopt.

Adolescent beliefs toward family formation, including ideal time to start childbearing, are of particular interest to adolescent health practitioners because of their strong link to future fertility behaviors among this group [17, 18]. While there is limited empirical evidence linking ideal age at first birth to actual fertility in LMICs, two studies in the United States found that the desired timing of first birth measured among adolescents ultimately predicted the fertility behavior among these adolescents several years after the initial data was collected [17, 19]. Understanding adolescent beliefs on when to start childbearing could help practitioners design educational programs during this period that could enhance positive beliefs regarding childbearing, which will translate to positive SRH behaviors in later years.

Furthermore, because gender attitudes formed during a variety of SRH behaviors and aadolescence shape the life trajectories of adolescent girls, including their SRH behaviors [5], it is important to understand and address unequitable gender attitudes such as gender differentiated parental control of adolescent behaviors before they crystallize and influence their SRH behaviors. Parental control of adolescent behaviors varies by the sex of the adolescent as parents often have different behavior expectations for boys and girls, an attitude that is deeply rooted in the prevailing social norms that socialize boys differently from girls [20, 21]. The greater restrictions placed on girls’ behavior than boys could reduce their ability to express themselves freely and develop life skills such as seeking information to address their SRH concerns [16, 22]. A global systematic review found that gender stereotypes and different expectations about appropriate sexual behavior for boys and girls influence sexual decision-making among adolescents [23].

### Agency of adolescent girls

The last decade has witnessed an increased interest in the SRH needs and the empowerment of adolescent girls, mostly stemming from the Sustainable Development Goals (SDGs) [24]. For example, a focus on specific aspects of adolescent sexual and reproductive health and rights such as contraception, adolescent birth rate and SRH information and education are key targets of the SDGs [25].

While most of the earlier investment in adolescent SRH have been targeted at programs largely focused on expanding access to and improving quality of health care services [26, 27], there is an increased focus on interventions to enhance adolescent girls’ agency.

Agency, the capacity to make decisions about one’s own life and act on them to achieve a desired outcome, free of violence, retribution, or fear, is a critical element in achieving healthy and positive SRH outcomes throughout the life course [28, 29]. Agency is often construed as an important part of women and girls’ empowerment as it is enacted when women and girls use their resources to make key decisions about their lives [28, 30–32].

A growing evidence base indicates that adolescent girls’ agency is related to a variety of SRH behaviors and antecedents, such as communication and norms [29, 33–35]. Adolescent girls with a perceived high level of agency (often measured as decision-making, freedom of movement, voice and gender equitable attitudes) were found to be at lower risk for early marriage, unwanted or mistimed pregnancy and recent childbearing [35]. Cross-sectional studies conducted among women and girls also found that higher level of agency was associated with higher contraceptive use [36–38], fewer births and longer birth intervals [39, 40] .

To our knowledge, the existing literature on the relationship between agency and SRH outcomes among very young adolescent (VYA) girls is limited as much of the existing quantitative research to date has focused on sexual activities among women or older adolescent girls aged 15–19 years [37, 38, 40, 41], mostly ignoring younger adolescent girls whose SRH skills are nascent as they have likely not initiated sexual activities.

### Role models and their influence on adolescent girls SRH

Beyond the influence of agency on SRH outcomes, the presence of role models within and outside households also plays a critical role in shaping the SRH behaviors of adolescent girls. The presence of a role model can improve SRH outcomes by developing girls’ aspirations for school completion and increasing possibilities for income generation [16, 26]. Despite the general acknowledgement of the positive influence of role models on adolescent SRH behaviors, there continues to be a dearth of empirical research to support this relationship. Most of the existing research is mostly qualitative in nature and focuses on the adolescent - role model interaction as an effective programmatic strategy in providing adolescent SRH services [16, 42, 43].

Additionally, most of the available quantitative studies on the relationship between role models and adolescent health outcomes have either largely been conducted in the United States [44–47] or limited to educational and other psychosocial outcomes [48–52]. As the potential significance of role models in improving adolescent SRH outcomes has been acknowledged, a more complete understanding of role models and their relation to SRH behavior is needed to inform and tailor adolescent SRH programs.

Using data that was collected as part of the Gender and Adolescent: Global Evidence (GAGE) baseline study in Ethiopia [53], this study examined two primary aims. The first aim was to determine the relationship between agency and two SRH outcomes – ideal age at childbirth and attitude towards gender-differentiated parental control of adolescent behaviors – among two cohorts of adolescent girls (10–12 and 15–17 years) in Ethiopia, using multivariate regression models. We hypothesized that agency is positively associated with ideal age at childbirth and negative attitude towards gender-differentiated parental control of adolescent behaviors.

The second aim was to assess the relationship between the presence of a role model and role model types on the two SRH measures, hypothesizing that the presence of a role model and having a familial role model type are associated with higher ideal age at childbirth and negative attitudes towards gender-differentiated parental control of adolescent behaviors. This study examined the two SRH outcomes – ideal age at childbirth and attitude towards gender-differentiated parental control of adolescent behaviors – among the adolescent girls because over two-thirds of the study sample are very young adolescent girls (10–12 years) whose SRH behaviors are nascent and therefore more important to understand the ideals and attitudes that influence their SRH trajectories.

## Methods

### Sample description

This study utilized 2017–2018 data from Ethiopia collected as part of the GAGE study [53]. GAGE is a longitudinal study exploring the gendered transition of adolescents in six LMICs, including Ethiopia (for more information on GAGE, see the GAGE Consortium, 2017 & 2019) [54, 55]. For this analysis, we focused on 3,028 never-married, in-school adolescent girls aged 10–12 and 15–17 years (Table 1).

The sample involved randomly sampled adolescents from rural and urban communities from three regions in Ethiopia: Afar, Amhara and Oromia, and Dire Dawa city administration. Amhara and Oromia were selected as regions of interest in the GAGE study because these regions make up over half of the Ethiopia population and are regions where child marriage is most prevalent among girls [56]. The median age of first marriage among women aged 20–24 years in the two regions is below 18 years [6]. Afar was also selected as a region of interest because the population is mainly pastoral and highly marginalized, which presents unique challenges for adolescents in accessing services [56].

The rural sites within each of the three regions were selected based on vulnerability criteria, including geographical areas with high rates of child marriage, as well as those that were economically disadvantaged and/or food insecure [54]. The rural sample was selected from 175 randomly selected sub-districts from three zones (South Gondar, East Hararghe and Zone 5).

The three urban sites selected (Batu, Debre Tabor and Dire Dawa) were within the proximity of the rural sites, allowing for better urban-rural comparisons. Batu and Dire Dawa, as a migration hub and migration corridor to the Middle East respectively, provide insights into new forms of employment, a major focus of the GAGE study. The urban sample was selected from 62 randomly selected neighborhoods from three locations (Batu, Debre Tabor and Dire Dawa) [56]. The younger cohort (10–12) sample was drawn from both rural and urban sites, while the older cohort (15–17) sample was only drawn from urban sites (one of the sites, Batu, had only the older cohort).

### Predictor variables

The primary independent measures explored in this paper are agency and role model characteristics. An agency six-item scale validated within the study sample was used to measure agency [57]. The six-item scale represents two domains, decision-making and mobility. The first three items of the scale represent decision-making on sexual and reproductive health issues. The respondents were asked about the amount of say they believed they had on when to marry, who to marry, and who to be friends with. The responses to these variables include: 1- None at all; 2- Not much; 3- A little bit; and 4- A great deal of say. The last three items of the agency scale represent mobility, and respondents were asked if they needed permission to go to the market; visit the homes of relatives, friends or neighbors; and go to a place in the community where they feel comfortable to meet with friends. The response to these questions was binary, yes (0) or no (2). We summed the 6 items into an agency scale, with an ordinal alpha of 0.96 (see Ogunbiyi et al. for more details) [57].

We also explored two specific characteristics for role models, the presence of a role model and role model type. For the presence of a role model, respondents were asked if there was someone they respect, follow, look up to, or want to be like. Respondents with role models were coded as yes or ‘1’, while those without role models were coded as a no ‘0’. Respondents with role models were further asked about the identity of the role model with response options including family members (coded as ‘1’), friends (coded as ‘2’), teachers, community leaders and other community members (coded as ‘3’) and famous individuals (coded as ‘4’). For the analysis on role model type, respondents with no role model were assigned ‘0’.

### Outcome variables

We explored two SRH outcome measures for adolescent girls - ideal age at childbirth and attitude towards gender-differentiated parental control of adolescent behaviors. The respondents were asked the ideal age they would like to start having children and their response was recorded in years. For the attitude towards gender-differentiated parental control of adolescent behaviors variable, the respondents were asked if they agree, partially agree or disagree that families should control their daughters’ behaviors more than their sons’. Agreement and partial agreement with the statement were coded as a ‘0’, and disagreement with the statement was coded as a ‘1’. Disagreement with the statement refers to attitude towards gender-differentiated parental control of adolescent behaviors. We referred to older ideal age at childbirth and a negative attitude towards gender-differentiated parental control scale as positive SRH outcomes in this study.

### Data analysis

Data analysis was conducted using STATA Version 15.1 (Stata Corporation, College Station, TX). We estimated relative frequencies of all items to assess their completeness and distributions. To minimize missing outcome data in the final analytic sample, we recoded the ‘don’t know’ response option representing a negligible proportion of the responses to the ideal age at childbirth variable (not more than 5%) to the mean of the observations [58]. Furthermore, we excluded the response option ‘God’s will or no preference’ from the final analytic sample as this option represented less than 2% of observations.

We dichotomized the continuous scores of agency at the median to identify adolescents with “high” or “low” agency in some of the descriptive statistics. We limited the age comparison of the predictors and outcome variables to two locations with both younger and older cohort of the adolescent girls (Debre Tabor in Amhara region and Dire Dawa). We also limited the rural/urban comparison to the younger cohort since the older cohort were only selected from urban settings.

We explored t-tests to compare ideal age at childbirth and agency within the two age cohorts and residence (rural vs. urban), and we applied chi square tests to compare the attitude towards gender-differentiated parental control of adolescent behaviors and role model characteristics of the girls by their age cohort and residence. Additionally, we tested the difference in the ideal age at childbirth by agency profile (low or high) and role model presence (yes or no) with t-tests, while we tested the difference in the proportion of girls with a negative attitude towards gender-differentiated parental control of adolescent behaviors by their agency profile and role model presence with chi square tests.

We applied one way analysis of variance (ANOVA) to identify significant differences in the ideal age at childbirth by role model types, and subsequently performed Tukey honest significant difference post hoc test to determine significant differences between pairs of group means of ideal age at childbirth.

Furthermore, we applied ordinary least squares and logistics regression models to explore the associations between the predictor variables and the SRH outcomes. Specifically, we applied ordinary least squares regression models to explore the associations between the standardized agency scale, role model characteristics and ideal age at childbirth among the adolescent girls.

Additionally, we conducted logistic regressions to identify the relationship between agency, role model characteristics and the attitude towards gender-differentiated parental control of adolescent behaviors. We ran three separate models to identify the individual main effect of each of the predicators (agency, role model presence, and role model type) on the outcome. The regression models are expressed as:

IA_*i*_ = 𝛼+ 𝛽_1_P_i_ + 𝛾𝑋′_*i*_+𝜀_*i*c_

Where IA_*i*_ is the outcome of interest (ideal age at childbirth) for girl *i*, P_ji_ is the key predictor variable (agency, role model presence, or role model type), and 𝑋′_*i*_ is a vector of controls. We clustered the standard errors (𝜀_*i*_) at the sub-kebele (community) level and used sampling weights to ensure that the results represent the target population in the study area  [56]. 𝑋′_*i*_ includes a set of covariates that may be associated with the outcomes of interest and likely correlated with independent variables of interest. The covariates include: three standardized variables of age and location given that the older girls were only sampled from urban households (young and rural, young and urban, and old and urban), household characteristics - household total asset score (a summation of 14 asset variables collected in both rural and urban locations, listed in appendix 2), literacy status of the household head, living in a female-headed household- and social norms measured among the female caregivers (generated from 12 norms statements described in detail in the appendix 1). These controls were selected based on controls used in similar studies in Ethiopia [33, 59].

Missing data were less than 10% for the fully specified regression models (OLS and logistics regression), and sensitivity analysis (available upon request) suggested that the data were ‘missing at random’ so these observations were dropped from the regression models. For each predictor, we first explored bivariate associations (model 1) before investigating multivariate associations with the control variables (model 2).

## Results

Table 1 describes the study sample. A total of 3,028 adolescent girls aged 10–12 and 15–17 years were included in this analysis. The average household asset score of the girls was 3.68 (± 2.27). Less than half (45.2%) of the girls were from households with literate household heads, while less than a third (20.3%) were from female-headed households. The average score of the gender norms measured among the female caregiver of both the younger and the older girls was 17.9 (+ 9.6), measured on a scale ranging from 0 to 36, with higher scores representing more gender equitable response.

**Table 1:** Characteristics of the selected sample

Characteristics	Frequency
Age 10–12 15–17 Total	2,453 (81.0) 575 (19.0) 3,028
Residence Urban Rural Total	966 (31.9) 2,062 (68.1) 3,028
Household asset score (0–14, higher scores are wealthier households)	3.68 (+ 2.27)
Household head is literate No Yes Total	1,650 (54.82) 1,360 (45.18) 3,010
Living in a female-headed household No Yes Total	2,411 (79.73) 613 (20.27) 3,024
Female caregiver gender norms (0–36, higher scores are more gender equitable)	17.85 (± 9.56)

*Notes*: This table summarizes the frequency distribution (and proportion) of relevant demographic characteristics of the adolescent girls included in this study from the Gender and Adolescence: Global Evidence (GAGE) Ethiopia baseline quantitative survey. This sample is restricted to randomly never-married, in-school adolescent girls with age data. The rural sample consists of only the younger cohort of adolescent girls (10-12years). There are small differences in sample sizes across demographic characteristics. The sample size at the bottom of each column reflects the maximum sample size for that sub-sample

The mean ideal age at childbirth was 25.9 years (± 4.6), with no marked difference in the older and young cohort (26.94 (± 3.24) and 27.17(± 3.99), respectively (Table 2). Among the younger cohort, girls living in urban locations reported significantly older ideal age at childbirth compared to their counterparts living in rural settings − 27.17 (± 3.99) and 25.40 (± 5.06), respectively.

**Table 2:** Ideal age at childbirth, Attitude, Agency and Role Model Characteristics by Age and Location

Variable	Overall (mean (SD) or n (%))	10–12 years (Urban)	10–12 years (Urban)	15–17 years (Urban)	15–17 years (Urban)
Ideal age at childbirth	25.9 (± 4.6)	25.40 (± 5.06)	27.17 (± 3.99)***	27.17 (± 3.99)	26.94 (± 3.24)
Attitude towards gender-differentiated parental control Agree Disagree Total	2,322 (76.68) 706 (23.32) 3,028	1,568 (76.04) 494 (23.96) 2,062	304 (77.75) 87 (22.25) 391	304 (77.75) 87 (22.25) 391	276 (74.59) 94 (25.41) 370
Agency (n = 3,028)	10.29 (± 3.48)	10.01 (+ 3.43)	10.52 (+ 3.21)**	10.52 (± 3.21)	11.60 (± 3.64)***
Has a role model No Yes Total	1,765 (58.37) 1,259 (41.63) 3,024	1,413 (68.66) 645 (31.34) 2,058	163 (41.69) 228 (58.31)*** 391	163 (41.69) 228 (58.31) 391	121 (32.70) 249 (67.30)* 370
Role model type None Family Friends Teachers/other community members Someone famous Total	1,765 (62.37) 266 (9.40) 250 (8.83) 370 (13.07) 179 (6.33) 2,830	1,413 (71.47) 113 (5.72) 150 (7.59) 273 (13.81) 28 (1.42) 1,977	163 (47.80) 53 (15.54) 42(12.32) 38 (11.14) 45 (13.20)*** 342	163 (47.80) 53 (15.54) 42 (12.32) 38 (11.14) 45 (13.20) 341	121(36.89) 60 (18.29) 40 (12.20) 41 (12.50) 66 (20.12)* 328

*Notes*: This table summarizes the frequency distribution (and proportion) of ideal age at childbirth, attitude towards gender-differentiated parental control of adolescent behaviors, agency and role model characteristics by age and location (rural/urban). The age comparison was limited to two urban locations (Debre Tabor and Dire Dawa) with the two age cohorts (maximum sample = 761). The location comparison was restricted to the younger cohort (maximum sample = 2,453) given that the older cohort were only selected from urban locations. There are differences in sample sizes across the variables. The sample size at the bottom of each cell reflects the maximum sample size for that sub-sample. *p < .05 **p < .01 ***p < .001

Less than a quarter of the girls (23.3%) expressed a negative attitude towards gender-differentiated parental control of adolescent behaviors, that is, they disagreed with the attitude statement that families should control their daughters’ behavior more than their sons. In the two urban locations with the younger and older cohorts, older girls expressed a negative attitude towards gender-differentiated parental control of adolescent behaviors compared to the younger girls (25.4% and 22.3%, respectively), this difference was however not significant. Among the younger cohort, there was no significant difference in the proportion of girls that expressed a negative attitude towards gender-differentiated parental control of adolescent behaviors in rural and urban settings (24.0% and 22.3%, respectively).

Measured on a 0–18 scale, the mean agency score was 10.29 (± 3.48), with older girls in urban areas reporting significantly higher level of agency than the younger cohort in urban areas. (11.60 (± 3.64) and 10.52 (± 3.21), respectively). Among the younger cohort, girls living in urban settings reported significantly higher level of agency than their counterparts living in rural settings (10.52 (+ 3.21) and 10.01 (+ 3.43), respectively).

Nearly 42% of the girls had a role model (someone they respect or look up to), with significant age differential in urban areas– over two-thirds of the older cohort compared to 58.3% of the younger cohort. More younger girls in urban settings reported having a role model compared to younger girls living in rural settings (58.5% and 31.3% respectively). Teachers and other community members, including community leaders and girls’ club leaders were the most reported role model type (13.1%), while famous individuals such as political figures or celebrities were the least cited role model type (reported by 6.3% of the girls). While family members were the most cited role model types by the younger cohort (15.5%) in urban areas, famous individuals were the most cited role model type by the older cohort (20.1%) in urban areas.

Table 3 shows the distribution of the two outcome variables by the level of agency and role model characteristics. The ideal age at childbirth differs significantly among girls with low and high agency, 25.6 years (± 4.6) and 26.1 years (± 4.7), respectively. Similarly, girls with role models had significantly higher ideal age at childbirth compared to those without a role model (26.6 years (± 4.3) compared to 25.4 years (± 4.8)). Girls whose role model are famous individuals, family members or friends reported significantly higher ideal age at childbirth (27.4, 27,2 and 26.9 years respectively) compared to those without a role model (25.4 years). Furthermore, girls with high agency profile expressed a negative attitude towards gender-differentiated parental control of adolescent behaviors compared to those with lower agency profile (25.4% and 21.7% respectively).

**Table 3:** Ideal age at childbirth and Attitude by Agency Profile and Role Model Characteristics

Variable	Ideal age at childbirth (mean (SD))	Attitude towards gender-differentiated parental control (n (%))	
		Agree	Disagree
Agency Low High	25.58 (± 4.64) 26.12 (± 4.65)**	1,152 (78.26) 970 (74.62)	320 (21.74) 330 (25.38)*
Has a role model No Yes	25.40 (± 4.81) 26.56 (± 4.32)***	1,364 (77.28) 954 (75.77)	401 (22.72) 305 (24.23)
Role model type None Family Friends Teachers/other community members Someone famous	25.39 (± 4.81) 27.20 (± 4.28)*** 26.90 (± 4.56) *** 25.58 (± 4.50) 27.39 (± 3.22)***	1,364 (77.28) 204 (76.69) 181 (72.40) 289 (78.11) 129 (72.07)	401 (22.72) 62 (23.31) 69 (27.60) 81 (21.89) 50 (27.93)

*Notes*: This table summarizes the frequency distribution (and proportion) of the ideal age at childbirth and attitude towards gender-differentiated parental control of adolescent behaviors by agency profile and role model characteristics. *p < .05 **p < .01 ***p < .001

Table 4 shows a positive and significant association between agency and the two SRH outcomes – a 1SD increase in agency is associated with an increase of 0.23 year in ideal age at childbirth, given the other variables (age, location, household characteristics, and social norms) are held constant in the model. Further, a 1SD increase in agency score increases the odds of disagreeing with the negative attitude towards gender-differentiated parental control by 18%. This suggests that girls with higher agency do not support gender-differentiated parental control of adolescent behaviors.

**Table 4:** Relationship between Agency, Ideal age at childbirth and attitude towards gender-differentiated parental control

	Ideal age at childbirth (β)		Attitude towards gender-differentiated parental control (OR)	
	Model 1	Model 2	Model 1	Model 2
Standardized scale of agency	0.39***	0.23**	1.25***	1.18***
Older and Urban (ref) Younger and rural Younger and urban		-2.32** -1.77**		0.12*** 0.07***
Household asset score		0.21***		1.01
Female-headed household No (ref) Yes		0.39**		1.27**
Literacy status of household head Not literate (ref) Literate		0.51*		1.00
Caregiver’s social norm		0.09**		1.12***
Sample size	2,658	2,641	3,028	3,010
Adjusted/Pseudo R2	0.0072	0.0403	0.0081	0.0426

*Note*: The table shows the results of the bivariate (model 1) and multivariate (model 2) relationship between standardized agency scale and the two SRH outcomes- ideal age at childbirth and attitude towards gender-differentiated parental control of adolescent behaviors. Control variables included in the models are household asset score, literacy status of the household head, living in a female-headed household; caregiver social norms (didn’t add caregiver gender attitudes because it highly strongly correlated with norms (r = .92), combined variables for age and location (young & rural, young & urban and old & urban). The R^2^ for the logistic regression models is the pseudo R^2^ .*p < .05 **p < .001 ***p < .0001

Table 5 shows a positive and significant association between having a role model and ideal age at childbirth - having a role model is associated with an increase of 0.77 year in ideal age at childbirth. There was no significant association of having a role model on the odds of both disagreeing with the negative attitude towards gender-differentiated parental control of behaviors.

Further, there was a significant relationship between some role model types and ideal age at childbearing- compared to reporting no role model, having family members, friends and famous individuals as the role models is associated with an increase of 1.45 years, 1.32 years and 1.01 years in ideal age at childbirth, respectively. Despite teachers and other community members being the most cited role model types, there was no significant difference in ideal age at childbirth and the girls’ attitude towards gender-differentiated parental control when the role model is a teacher or other community member compared to no role model. Also, compared to reporting no role model, identifying famous individuals as the role model increases the odds of disagreeing with the negative SRH attitude statement by 57%, given the other variables are held constant in the model.

**Table 5:** Relationship between Role model characteristics, Ideal age at childbirth and attitude towards gender-differentiated parental control

	Ideal age at childbirth (β)		Attitude towards gender-differentiated parental control (OR)	
	Model 1	Model 2	Model 1	Model 2
Panel 1: Role model presence				
Has a role model	1.18***	0.77**	1.03	1.00
Older and Urban (ref) Younger and rural Younger and urban		-2.14*** -1.73**		0.12*** 0.06***
Household asset score		0.21***		1.01
Female-headed household No (ref) Yes		0.37**		1.28**
Literacy status of household head Not literate (ref) Literate		0.48*		1.02
Caregiver’s social norm		0.09**		1.12***
Sample size	2,655	2,638	3,024	3,006
Adjusted/Pseudo R2	0.0159	0.0441	0.0000	0.0382
Panel 2: Role model characteristics				
	**Model 1**	**Model 2**	**Model 1**	**Model 2**
Role model characteristics None (ref) Family Friends Teachers/other community members Someone famous	1.94*** 1.72** 0.09 1.88***	1.45** 1.32* 0.13 1.01**	0.96 1.31 0.89 1.30*	0.98 1.11 0.88 1.57**
Older and urban (ref) Younger and rural Younger and urban		-2.15*** -1.88***		0.11*** 0.06***
Household asset score		0.19***		1.00
Female-headed household No (ref) Yes		0.42**		1.24**
Literacy status of household head Not literate (ref) Literate		0.48*		1.01**
Caregiver’s social norm		0.10***		1.12***
Sample size	2,482	2,465	2,830	2,812
Adjusted/Pseudo R2	0.0297	0.0511	0.0020	0.0418

*Note*: The table shows the results of the bivariate (model 1) and multivariate (model 2) relationship between role model characteristics and the two SRH outcomes- ideal age at childbirth and attitude towards gender-differentiated parental control of adolescent behaviors. Control variables included in the models are household asset score, literacy status of the household head, living in a female-headed household; caregiver social norms, combined variables for age and location (young & rural, young & urban and old & urban). The R^2^ for the logistic regression models is the pseudo R^2^. *p < .05 **p < .001 ***p < .0001

## Discussion

This is one of the first studies in a LMIC context to investigate the relationship between adolescent girls’ agency, role model characteristics and SRH behaviors; and the first study to the best of our knowledge to focus on a sample that is predominantly VYAs. Our findings confirm a positive relationship between agency and the SRH outcomes, giving credence to interventions that seeks to build agency as an enabler of positive SRH outcomes among adolescents in later years [16, 60, 61]. This study also contributes novel evidence by assessing the association between role model presence and characteristics and SRH outcomes, empirically confirming that the presence of role model and specific type of role model is associated with positive SRH outcomes.

First, we note that the average ideal age at childbirth of 25.9 years reported by the girls in this study differs considerably from the reality of adolescent girls in the country as the average age at first birth among women in the country is 18 years [62]. This finding suggests that adolescent girls in the study areas would generally aspire to delay childbirth by at least 8 years, an aspiration that is devoid of the reality of early childbearing and tends to be adjusted through adolescence to realities imposed by prevailing social norms and economic constraints which drives early marriage and childbirth in Ethiopia [63].

The considerable variation in ideal age at childbirth by location with higher age reported by girls in urban locations compared to girls in rural locations, confirms existing literature on location exerting an important influence on SRH outcomes of adolescent girls in Ethiopia [64, 65]. Some researchers have linked the better SRH outcomes reported among adolescent girls living in urban settings such as early marriage and higher age at childbirth to better access to SRH information and services [65–68].

The finding of a positive relationship between agency and the two SRH outcomes support the manifestation of agency as an important determinant of critical decisions such as SRH issues and resources to act [28–30, 32, 69–71]. It is understandable that girls who feel that they have more control over their lives will also demonstrate better SRH antecedents and by extension SRH behavior. This finding aligns with findings from a similar study in Zambia which reported that over time, adolescent girls with higher agency were least likely to experience negative SRH outcomes such as early marriage and recent childbearing [35].

The positive association between agency and the SRH outcomes also confirms the findings of a study among adolescents in rural Tanzania which reported that perceived behavior control (a similar construct to agency that captured perceived self-efficacy and perceived controllability over SRH-related decisions), and the concept of empowerment predicted SRH intention (to use condom) and actual SRH behavior (condom use) [72].

The higher ideal age at childbirth and negative attitude towards gender-differentiated parental control of adolescent behaviors reported among girls with higher agency can be explained within the context of agency being influenced by social norms in a specific context [33]. In most LMICs, social norms on SRH issues often creates a culture of silence for adolescent girls to obtain information or express their SRH concerns to adults within their communities [16]. However, girls with higher agency are likely able to acquire skills such as critical awareness, problem solving, self-efficacy and communication skills to help navigate the restricted norms in their communities [22]. With these life skills, they are better able to express concerns related to their lives, including SRH concerns and seek SRH information [22].

Furthermore, girls with enhanced agency express attitudes of autonomy, responsibility and are risk averse, which in turn encourages them to protect their own sexual and reproductive health [22]. Such girls also have aspiration for a future outside of childbearing and hold more positive SRH attitudes when compared to those with lower perception or control over their decisions and freedom of movement [43].

That girls with role models reported later ideal age at childbearing compared to their counterparts without role models support the social learning theory which purports that through observational learning, adolescents may adopt the behaviors of their adult role model, including their SRH values and behaviors [73]. This finding is consistent with other studies that found a positive association between role model presence and positive health outcomes among adolescents [44–47]. This finding suggests that the role model is modelling positive attitudes and behaviors that these girls have adopted as it is also possible for role models to model deviant health behaviors that can lead to negative influence on the adolescents’ health outcomes. Further research is needed to understand the exact types of SRH behaviors being modelled by these role models in the Ethiopian context.

The findings also indicate differential influence of role model types on the two outcomes of interest, suggesting that the type of role model matters in adolescent SRH programming. That most of the role models identified by the girls are not familial corroborates a Malawian study which found that adolescent girls often express a desire for role models or “outside experts” to provide SRH education and to promote an alternate vision to adolescent motherhood [43]. While parents and other family members of the adolescents are often perceived as the role models that influence the choices that these girls make about their own SRH behavior, having a role model outside of the family unit could give the girls a sense of future possibilities related to delayed childbearing [43,74]. A non-parental role mode with whom the girl has a personal link to may reflect the girls’ broader social network and her ability to establish relationships outside of her family [47].

Despite teachers and community members being the most cited role model types, there was no significant difference in ideal age at childbirth and attitude towards gender-differentiated parental control of adolescent behaviors reported by girls with role model type compared to those with no role model. The lack of association between having a teacher or community member as a role model and more equitable attitude towards gender-differentiated parental control of adolescent behaviors contrast previous findings of positive health behaviors among adolescents that identified teachers as their role model [75–77]. To understand the reason for this unexpected finding, further research is needed to explore the type of influence this role models have on various SRH behaviors (or antecedents) and to understand the types of behaviors modelled by teachers and other community members in the Ethiopian context. Considering that most community-based programs to address adolescent SRH view teachers and community leaders as community assets that can enhance the SRH of adolescents, this finding suggests the need to carefully assess the behaviors of these role models prior to large scale implementation of such program strategy.

Our findings on familial role model suggest that the influence of this role model type depends on the SRH behavior (or antecedents) being assessed - while these role models confer no influence on attitude towards gender-differentiated parental control of adolescent behaviors compared to not having a role model, they confer the highest influence on ideal age at childbirth. Compared to the influence of other role model types, the highest influence of familial role models on ideal age at childbirth may be due to more frequent contact with familial role models, allowing familial role models more opportunities to have an influence on adolescents’ SRH outcomes. It is worth noting that these familiar role models are in most cases not the parents of the girls, but siblings and extended family members like uncles and aunts. More research is needed using wider (and proximate) SRH behaviors to distil how familial role model influence adolescent SRH outcomes. Notwithstanding the mixed finding, the evidence from this study suggests the need to engage non-parent, familial members as role models when planning SRH programs for adolescent girls.

The finding that having a peer as role model (compared to no role model) increases the ideal age at childbirth significantly suggests that peers can also have a positive influence on adolescent girls SRH and could be useful asset for adolescent girls’ program planners. That famous individuals present the lowest (ideal age at childbirth) to highest (attitude towards gender-differentiated parental control of adolescent behaviors) protective influence on the girls SRH (compared to no role model) deviates from the cumulative, but limited evidence on the negative influence of influential individuals on adolescent SRH [75]. This finding suggests that there it is still better for the girls to have an individual they look up to that is someone they don’t have access to rather than not having a role model at all. It further corroborates the centrality of role model influence on positive youth development.

Beyond contributing to the literature, the findings of this study have important implications for public health practice, particularly related to interventions that seeks to build agency as an enabler of positive SRH outcomes among adolescents in later years.

Given that adolescent girls with higher level of agency reported later ideal age at childbirth and negative attitude towards gender-differentiated parental control of adolescent behaviors, interventions designed to foster positive SRH outcomes among adolescent girls should consider developing this capability – agency – as a core program component, particularly in settings where adolescents generally have low to moderate levels of agency.

Considering the variation of the attitude towards gender-differentiated parental control by age with older girls expressing more gender equitable attitudes compared to younger girls, SRH interventions among adolescents should particularly target younger adolescents who appears to have already internalized the prevailing restrictive norms around SRH in their communities.

The positive association between role model presence and ideal age at childbirth such that having a role model was associated with an increase of 0.77 year in ideal age at childbirth suggests that interventions targeted at adolescent girls should consider including role modelling as a critical program component. Such interventions should also plan to measure actual fertility or SRH behaviors among the girls both in the short and long term.

With respect to the differential association between role model type and ideal age at childbirth *—*having a familial, peer and famous individual role model were associated with an increase of 1.45years, 1.32years and 1.01years in ideal age at childbirth, respectively *—* adolescent interventions must move beyond engaging any type of role models to intentionally engaging familial, peers and famous individuals in these programs.

Further, given that most of the familial role models identified in this study were not parents, but rather immediate and extended family members such as siblings, uncles and aunts, public health practitioners should consider that parents are not necessarily perceived as a role model among adolescent girls and that a non-parental role model with whom the girl has a personal relationship with may reflect her broader social network and her ability to establish relationships outside of her family [75]. Furthermore, positive adolescent development programs should leverage peer influence in engendering positive SRH behaviors among adolescent girls.

Considering that majority of the older girls identified famous individuals as their role models and this role model type was associated with negative attitude towards gender-differentiated parental control of adolescent behaviors, adolescent interventions with role model components should consider engaging famous individuals such as key political figures and sport icons to promote equitable gender norms, especially when the intervention is targeted at older adolescent girls.

Lastly, another practical implication of this study on adolescent SRH interventions is the absence of a positive association bet having a teacher or a community leader as a role model and ideal age at childbirth and attitude towards gender-differentiated parental control of adolescent behaviors. Since most community based adolescent SRH interventions consider teachers and community leaders as assets that can enhance the SRH of adolescents, this finding suggests the need for practitioners to re-evaluate this approach prior to large scale implementation of such program strategy as it might not be effective in some settings (such as the current study setting).

### Limitations

There are some important limitations in this study. First, the use of secondary data limited the scope of variables included in the analyses. The measures of SRH behaviors included in the analyses could have been expanded and further refined to capture proximate SRH behaviors among the adolescent girls, especially the older girls. Despite this limitation, data on the two SRH outcomes - ideal age at childbirth and attitude towards gender-differentiated parental control of adolescent behaviors - could shed light on young people’s perception to delaying childbirth, a critical SRH outcome, in line with the changing social norms in Ethiopia.

Second, the observed relationships between the predictor and outcome variables cannot be interpreted as causation and the exact direction of the relationships cannot be ascertained due to cross-sectional characteristics of the data. For example, the finding of a positive relationship between agency and the SRH outcomes could also reflect an interchangeable relationship between agency and the SRH outcomes as girls who have experienced negative SRH outcomes (such as early sexual debut and childbearing) may be detracted from enhanced agency [60]. A recent longitudinal study reported such bidirectional relationship between agency and SRH outcomes [35]. Further analysis using longitudinal data from the subsequent waves of the GAGE study can expound on the causal and directionality of the relationship between agency, role model characteristics and SRH behaviors as the girls develop more concrete SRH behaviors.

Third, we cannot rule out social desirability bias in self-reported SRH outcomes and agency indicators among the girls given the sensitive nature of the study and most respondents may refrain from stating the truth about their beliefs (attitudes) if they believe it portrays an unfavorable image of themselves. High level of social desirability in the data will greatly impact the reliability of critical measures such as the SRH outcomes and agency of the adolescent girls.

## Conclusion

Taken together, our findings suggest that critical adolescent capability – agency and asset – specific role models are linked to shaping SRH behaviors in the adolescent period in the context of rural and urban Ethiopia. This study contributes to the field by linking agency, a component of the broader concept of empowerment, to adolescent behaviors especially among young adolescent girls in Ethiopia who are still on a SRH growth trajectory. We conclude that further research, with longitudinal data and more specific SRH behavior measures, is needed to fully understand the role of agency and role model characteristics in shaping SRH trajectories during early adolescence.

### Electronic supplementary material

Below is the link to the electronic supplementary material.

Supplementary Material 1

## Data Availability

The datasets used for this study is from a publicly available website and can be accessed from the United Kingdom Data Archive - https://beta.ukdataservice.ac.uk/datacatalogue/studies/study?id=8597.
