# Supplementary material for: Agency and role models: do they matter for adolescent girls’ sexual and reproductive health?
Source: BMC Womens Health. 2023 Sep 27;23:515. doi: 10.1186/s12905-023-02659-8 (PMC10523787; doi:10.1186/s12905-023-02659-8)
Supplement: Supplementary file 1 — Supplementary Material 1 [file 12905_2023_2659_MOESM1_ESM.docx]

# Appendix 1: Measure of social norms of female caregivers

| **Measure** | **Indicator** | **Response Options** |
| --- | --- | --- |
| Social norms of female caregivers | - 1. Most men in my community are the ones who make the decisions in their home.   2. Most boys and girls in my community do not share household tasks equally.   3. Most people in my community expect men to have the final word about decisions in the home.   4. Adolescent girls in my community are more likely to be out of school than adolescent boys.   5. Girls in my community are sent to school only if they are not needed to help at home.   6. Most people in my community think that new people in my community threaten our jobs and values.   7. Most people in my community expect women to have the same chance to work outside the home as men.   8. Most people in my community think that violence between husbands and wives is a private matter and others should not interfere.   9. People in my community do not interfere in arguments between wife and husband even if they know violence is happening.   10. Most women in my community have the same chance to work outside the home as men.   11. Most families in my community control their daughters’ behaviors more than their sons’ to preserve the family’s reputation.   12. Most people in my community expect families to control their daughter’s behavior more than their sons to preserve the family’s reputation.   13. Most people in my neighborhood/community can be trusted.   14. Our culture makes it harder for girls to achieve their goals than boys. | 0 - Agree  1 - Partially agree  2 - Disagree |

Notes: The female caregiver social norms measure was coded such that respondents were assigned a ‘2’ if they agreed, a ‘1’ if they partially agreed and a ‘0’ if they disagreed with a favorable statement on gender equity. We reverse coded statements with a negative valence so that ‘2’ indicated a disagreement and ‘0’ indicated an agreement with unfavorable statements. We reversed coded ten statements that describe gender inequity (statements 1-6, 8-9, & 11-12) such that respondents that disagreed with each statement were assigned a ‘2’, those that partial agreed with each statement were assigned a ‘1’ and those that agreed with each statement were assigned a ‘0’. For four statements that describe gender equity (statements 7, 10, 13 & 14), we assigned a ‘2’ when the respondent agreed, a ‘1’ when the respondent partially agreed, and a ‘0’ when the respondent disagreed with the statement.

Appendix 2: Measure of household asset

| **Measure** | **Indicator** | **Response Options** |
| --- | --- | --- |
| Household asset score | Does the household own a [Item] ?  a. Mattress and/or bed  b. Working refrigerator or freezer  c. Working computer, laptop, or tablet  d. Working mobile phone  e. Working radio  f. Working television  g. Working bicycle  h. Working motorcycle, motor scooter or bajaj  i. Working car or truck  j. Working wrist watch / clock  k. Sofa set  l. Table  m. Chair  n. Plow | 0 – No  1 – Yes |

Notes: For household assets, we constructed this variable as a summation of 14 asset variables collected in both rural and urban locations. We assigned a ‘0’ when the household does not have the asset and a ‘1’ when the household has the asset. We then summed these 14 assets to obtain the household asset variable.
